# Protection against SARS-CoV-2 by BCG vaccination is not supported by epidemiological analyses

**DOI:** 10.1038/s41598-020-75491-x

**Published:** 2020-10-27

**Authors:** Janine Hensel, Kathleen M. McAndrews, Daniel J. McGrail, Dara P. Dowlatshahi, Valerie S. LeBleu, Raghu Kalluri

**Affiliations:** 1 Department of Cancer Biology, Metastasis Research Center, University of Texas MD Anderson Cancer Center, Houston, TX USA; 2 Department of Systems Biology, University of Texas MD Anderson Cancer Center, Houston, TX USA; 3 Feinberg School of Medicine, Northwestern University, Chicago, IL USA; 4 Department of Bioengineering, Rice University, Houston, TX USA; 5 Department of Molecular and Cellular Biology, Baylor College of Medicine, Houston, TX USA

**Keywords:** Health policy, Respiratory tract diseases, Health policy, Respiratory tract diseases

## Abstract

The Bacillus Calmette–Guerin (BCG) vaccine provides protection against tuberculosis (TB), and is thought to provide protection against non-TB infectious diseases. BCG vaccination has recently been proposed as a strategy to prevent infection with SARS-CoV-2 (CoV-2) to combat the COVID-19 outbreak, supported by its potential to boost innate immunity and initial epidemiological analyses which observed reduced severity of COVID-19 in countries with universal BCG vaccination policies. Seventeen clinical trials are currently registered to inform on the benefits of BCG vaccinations upon exposure to CoV-2. Numerous epidemiological analyses showed a correlation between incidence of COVID-19 and BCG vaccination policies. These studies were not systematically corrected for confounding variables. We observed that after correction for confounding variables, most notably testing rates, there was no association between BCG vaccination policy and COVD-19 spread rate or percent mortality. Moreover, we found variables describing co-morbidities, including cardiovascular death rate and smoking prevalence, were significantly associated COVID-19 spread rate and percent mortality, respectively. While reporting biases may confound our observations, our epidemiological findings do not provide evidence to correlate overall BCG vaccination policy with the spread of CoV-2 and its associated mortality.

## Introduction

Since the early 1920s, the use of the Bacillus Calmette–Guerin (BCG) vaccine has been implemented in several countries to prevent tuberculosis (TB), a disease caused by *Mycobacterium tuberculosis*. Infection with *Mycobacterium tuberculosis* primarily impacts lung function, and may lead to TB-related death. Immunization is done by exposing individuals to the *M. tuberculosis*-related, live, attenuated *Mycobacterium bovis* strain. The BCG vaccine thus offers a partial and possibly variable immunity against TB, and its off-target effect has also been linked to protection against other infectious diseases^1^. The mechanism of protection against a broad range of pathogens has been linked to epigenetic reprogramming of monocytes and natural killer cells which then enables innate immune cells to fight a broad range of pathogens^2^.

In most countries with high TB incidence, a universal BCG vaccination policy has been implemented, where children receive the BCG vaccine in early infancy. In some of these countries, when TB incidence dropped, the universal policy was discontinued. In addition, a few countries have never implemented a universal BCG policy due to low TB incidence and the possibility to retain use of the tuberculin skin test in order to assess current TB infections. In countries with active BCG vaccination programs, the variability in protection against TB could be due to differences in the BCG strains used in vaccine synthesis, vaccine batch differences, time of vaccination, previous exposure to mycobacterium, and distinct host haplotypes^3^. The BCG vaccine may offer protection for as long as 30–40 years post vaccination (as previously reported in Norway^4^), and possibly as long as 50–60 years, as reported in American Indians and Alaska Natives that participated in a BCG vaccine trial^5^. However, these studies also show a reduction in long-lasting protection which is in line with studies showing that the protective effect of the BCG vaccine is decreased over time (reviewed in^6^).

There is evidence from previous outbreaks that supports the hypothesis that BCG vaccination can protect against RNA and DNA viral infections (herpes and influenza) and other diseases such as asthma, as well as lowers yellow fever viremia^2,7,8^. Encouraging data also emerged from a recent double-blind, randomized clinical trial, showing that patients ≥ 65 years vaccinated with BCG had a reduced incidence of infections, in particular respiratory tract infections^9^. This study is in line with other previous reports showing a reduction in respiratory tract infections in the BCG vaccinated cohort^10,11^.

Preliminary epidemiological analyses have shown BCG vaccination is associated with reduced COVID-19 cases and mortality^12–17^. These observations have given support to the possibility of the BCG vaccine providing protection against infection with the novel coronavirus, SARS CoV-2 (CoV-2). In consideration for the potential protection of BCG vaccination against other infectious diseases including CoV-2 infection, seventeen clinical trials assessing the efficacy of BCG vaccination at preventing COVID-19 have been registered (Table 1). However, many of the epidemiological observations do not account for potential confounding variables, including socioeconomic factors, co-morbidities, and CoV-2 testing rate. In this study, we systematically analyzed whether the BCG vaccination policy of countries correlates with COVID-19 spread and mortality after adjusting for confounding variables.

**Table 1:** Registered clinical trials for COVID-19 related studies.

Clinical trial number	Title	Geographic location	Cohort Size	BCG strain	BCG vaccination policy
NCT04347876	Outcome of COVID-19 Cases Based on Tuberculin Test: Can Previous BCG Alter the Prognosis?	Egypt	100	NA	Current
NCT04350931	Application of BCG Vaccine for Immune-prophylaxis Among Egyptian Healthcare Workers During the Pandemic of COVID-19	Egypt	900	unknown	Current
NCT04461379	Prevention, Efficacy and Safety of BCG Vaccine in COVID-19 Among Healthcare Workers	Mexico	908	Tokio 172 strain	Current
NCT04379336	BCG Vaccination for Healthcare Workers in COVID-19 Pandemic	South Africa	500	Danish strain 1331, SSI, Denmark	Current
NCT04362124	Performance Evaluation of BCG Vaccination in Healthcare Personnel to Reduce the Severity of SARS-COV-2 Infection	Columbia	1000	BCG Liofilizada	Current
NCT04475302	BCG Vaccine in Reducing Morbidity and Mortality in Elderly Individuals in COVID-19 Hotspots	India	2175	Serum Institute of India	Current
NCT04369794	COVID-19: BCG As Therapeutic Vaccine, Transmission Limitation, and Immunoglobulin Enhancement (BATTLE)	Brazil	1000	Calmette Guerin bacillus	Current
NCT04327206	BCG Vaccination to Protect Healthcare Workers Against COVID-19 (BRACE)	Australia	10,078	Danish strain 1331, SSI, Denmark	Past
NCT04542330	Using BCG to Protect Senior Citizens During the COVID-19 Pandemic	Denmark	1900	Danish strain 1331, SSI, Denmark	Past
NCT04373291	Using BCG Vaccine to Protect Health Care Workers in the COVID-19 Pandemic	Denmark	1500	Danish strain 1331, SSI, Denmark	Past
NCT04384549	Efficacy of BCG Vaccination in the Prevention of COVID19 Via the Strengthening of Innate Immunity in Health Care Workers (COVID-BCG)	France	1120	unknown	Past
NCT04414267	Bacillus Calmette-guérin Vaccination to Prevent COVID-19 (ACTIVATEII)	The Netherlands	900	BCG vaccine Moscow strain 361–1; Serum Institute of India Pvt. Ltd	Never
NCT04417335	Reducing COVID-19 Related Hospital Admission in Elderly by BCG Vaccination	The Netherlands	2014	Danish strain 1331, SSI, Denmark	Never
NCT04537663	Prevention Of Respiratory Tract Infection And Covid-19 Through BCG Vaccination In Vulnerable Older Adults (BCG-PRIME)	The Netherlands	5200	Danish strain 1331, SSI, Denmark	Never
NCT04328441	Reducing Health Care Workers Absenteeism in Covid-19 Pandemic Through BCG Vaccine (BCG-CORONA)	The Netherlands	1500	unknown	Never
NCT04534803	BCG Against Covid-19 for Prevention and Amelioration of Severity Trial (BAC to the PAST)	USA	2100	Tokyo-172 Strain	Never
NCT04348370	BCG Vaccine for Health Care Workers as Defense Against COVID 19 (BADAS)	USA	1800	BCG Tice strain	Never

## Methods

We collected information for BCG vaccination policies, BCG strains, TB incidence and BCG coverage across all countries from the BCG World Atlas (https://www.bcgatlas.org/). For analysis of countries with past BCG vaccination policy, countries with a year BCG was introduced and stopped were included. The BCG World Atlas was last updated in 2017. Data of total COVID-19 cases, new COVID-19 cases, first reported COVID-19 cases, mortality, and testing rates for each country was obtained June 10th, 2020 from the COVID-19 Data Repository by the Center for Systems Science and Engineering (CSSE) at Johns Hopkins University (https://github.com/CSSEGISandData/COVID-19). Total population, population density (population/km^2^), fraction of population over 65, fraction of the population that lives in urban areas, per capita GDP, smoking rates, diabetes prevalence, and cardiovascular disease related mortality were obtained from Our World in Data (https://ourworldindata.org). World maps were generated using: https://mapchart.net/world.html.

Data were analyzed using Matlab R2016a. For COVID-19 spread rates, data were modeled to an exponential growth equation, *Cases* ~ *Cases0*∙exp(*k*_spread_∙*t*), where *Cases* is cases per million inhabitants, *Cases0* is the number of cases per million inhabitants at the initial time point, *k*_spread_ is the COVID-19 spread rate, and *t* is the time point in days. The initial time point was considered as when countries exceeded 1 case per million inhabitants, and the spread rate was fit over the first 21 days after COVID-19 cases reached 1 per million inhabitants. Univariate and multivariate regression analysis was performed on COVID-19 spread rates and percent mortality, defined as deaths per total COVID-19, cases using the fitglme function. All confounding variables were z-normalized for equivalent scaling, and p-values corrected using Benjamini and Hochberg procedure for univariate comparisons. For primary analyses, BCG vaccination (BCG Vac.) was treated as continuous variable divided between 3 discrete levels, 0 for never universal BCG vaccination policy, 1 for universal BCG vaccination policy in the past, and 2 for current universal BCG vaccination policy. Additional analyses were performed using two discrete levels for BCG vaccination policy, dividing into never or past universal BCG vaccination policy compared to current BCG vaccination policy. For some comparisons, countries were limited to those with testing rates of 10 tests per thousand and greater, as specified in the results.

## Results

As of June 13th, 2020, 7,713,571 CoV-2 infected individuals have been confirmed across the globe. Interestingly, 17 clinical trials are currently registered to evaluate BCG vaccination for COVID-19 on https://clinicaltrials.gov (Table 1). Seven trials are initiated in countries that currently have a universal BCG policy in place (Egypt, Mexico, South Africa, Columbia, India and Brazil; Table 1). Four trials are conducted in countries that had a universal BCG policy in the past (Australia, Denmark, and France) and 6 trials are in the Netherlands and USA, two countries that never had a universal BCG policy (Table 1). The largest study, based on numbers of participants, is underway in Australia to study the effects of BCG vaccination on COVID-19 incidence, duration and severity of disease.

**Figure 1:**
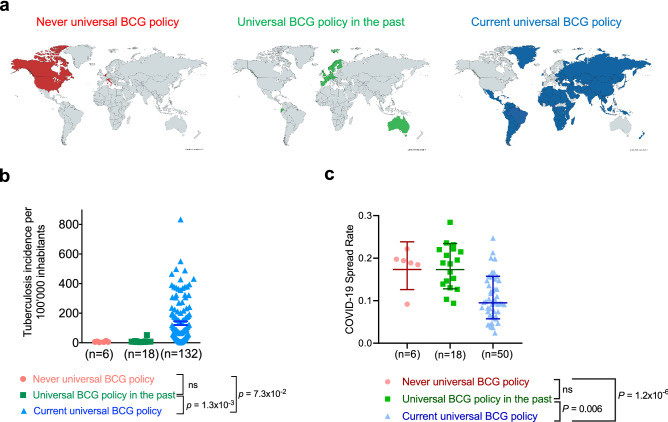
COVID-19 spread rate in countries with distinct national BCG vaccination policy. (**a**) World maps show countries that never had a national BCG vaccination policy (in red), countries that had a universal BCG vaccination policy in the past (in green) and countries that currently have a universal BCG vaccination policy (in blue). (**b**) TB incidence per 100,000 inhabitants shown as mean ± SEM for all three groups of BCG policies (never (red), past (green) and current (blue) BCG vaccination policy). One-way ANOVA with Tukey posttest was performed. (**c**) CoV-2 spread rate is shown for all three groups of BCG policies (never (red), past (green) and current (blue) BCG vaccination policy). Data shown as median ± interquartile range. Maps were generated using the following website: https://mapchart.net/world.html.

Intrigued by the extensive number of clinical trials initiated, we wanted to analyze currently available data to test the association between BCG vaccination policy and CoV-2 spread rate and its associated mortality. BCG vaccination policy information is available for 158 countries (Supplementary Table S1). Belgium, Netherlands, Canada, Italy, Lebanon, and United States of America never had a universal BCG policy, yet all these countries offer vaccination for high risk subgroups (healthcare professionals and children with parents from high risk countries) (‘Never universal GCG policy’, Fig. 1a). 18 countries had a universal BCG policy in the past, including many European countries, Australia, Ecuador, and Israel (‘Universal BCG policy in the past’, Fig. 1a). 134 countries currently have a universal BCG vaccination policy, including most countries in Central America, South America, Africa, and Asia (‘Current universal BCG policy’, Fig. 1a). First, we analyzed TB incidence in all countries with BCG policy information. Two countries with BCG policy information had no TB incidence numbers listed on the BCG World Atlas and therefore were not included in our analysis. Our analysis showed that countries with an active universal BCG policy have higher TB incidence as compared to countries that had a universal BCG in the past (Fig. 1b). We had complete data available for 74 countries to study the association of BCG vaccination policy and COVID-19 spread and mortality. The COVID-19 spread rate was significantly reduced in countries with current universal BCG policy when compared to countries with a never- or in the past-universal BCG policy (Fig. 1c).

The administered BCG vaccination strain did not account for a higher mortality per 100,000 inhabitants (Supplementary Figure S1a). Spain and Sweden had a high mortality per 100,000 inhabitants and both countries had a universal BCG policy in the past. Based on this observation we analyzed whether the year in which the BCG policy was discontinued correlates with mortality. We found that the mortality per 100,000 inhabitants was not correlated with the time the universal BCG policy was discontinued (Supplementary Figure S1b). Next, we analyzed mortality in countries with current universal BCG policy. There was no correlation observed in the mortality per 100,000 inhabitants and the time since implementation of the universal BCG policy (Supplementary Figure S1c). BCG coverage data was available for 51 countries which provides an estimate on access and adherence to BCG policy in each country (Supplementary Table S2). Except for Ukraine, which reported a coverage of 15–45%, most countries report a coverage higher than 90%.

**Figure 2:**
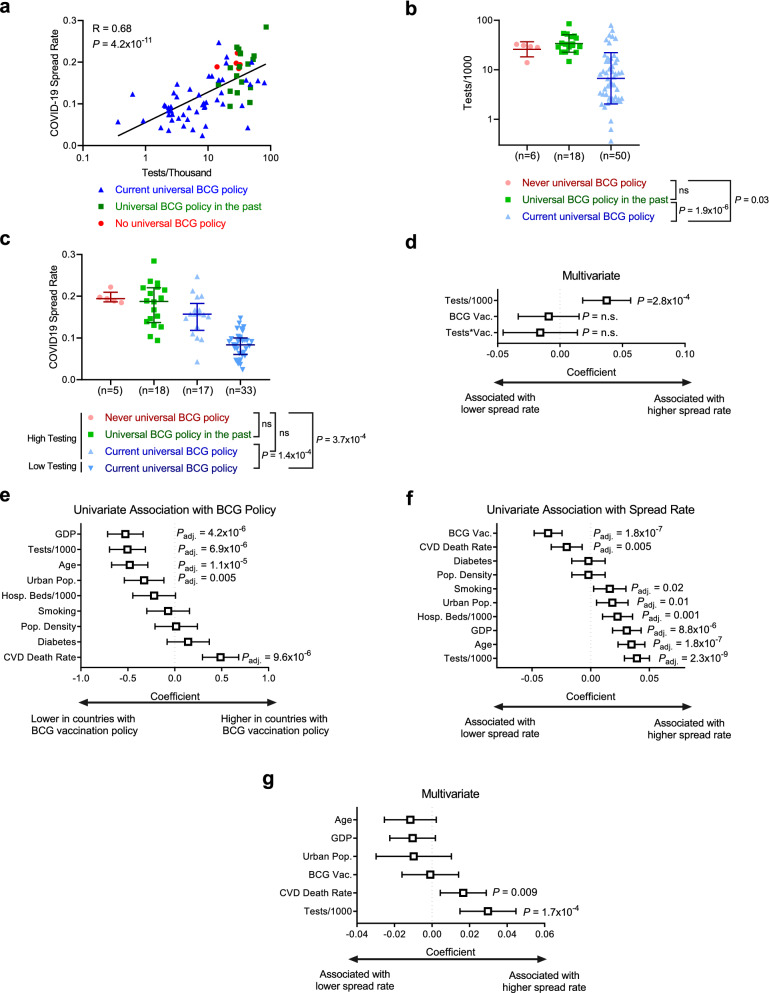
SARS-CoV-2 testing rates influence the observed benefit of BCG vaccination policy. (**a**) Correlation graph of total COVID-19 spread rate and tests per thousand inhabitants. Inset value is Spearman correlation coefficient (ρ). (**b**) Tests/1000 is shown for all three groups of BCG policies (never (red), past (green) and current (blue) BCG vaccination policy) comparing high and low testing rate. Data shown as median with interquartile range. Kruskal–Wallis with Dunn’s post-hoc test performed. (**c**) CoV-2 spread rate is shown for all three groups of BCG policies (never (red), past (green) and current (blue) BCG vaccination policy) comparing high and low testing rate. Data shown as median with interquartile range. Kruskal–Wallis with Dunn’s post-hoc test performed. (**d**) Multivariate regression analysis of CoV-2 spread rate shows the coefficients and *p values* for tests per 1000 inhabitants. (**e**) Univariate analysis of associations with BCG vaccination policy shows the coefficients and adjusted *p values* for cardiovascular disease (CVD) death rate, diabetes population density (pop. density), smoking rate, urban population (urban pop.), hospital beds per 1000 inhabitants, gross domestic product (GDP), age and tests per 1000 inhabitants. (**f**) Univariate analysis of associations with CoV-2 spread rate shows the coefficients and adjusted *p values* for cardiovascular disease (CVD) death rate, diabetes population density (pop. density), smoking rate, urban population (urban pop.), hospital beds per 1000 inhabitants, gross domestic product (GDP), age and tests per 1000 inhabitants. (**g**) Multivariate regression analysis of CoV-2 spread rate for variables significant in 2e and 2f.

**Figure 3:**
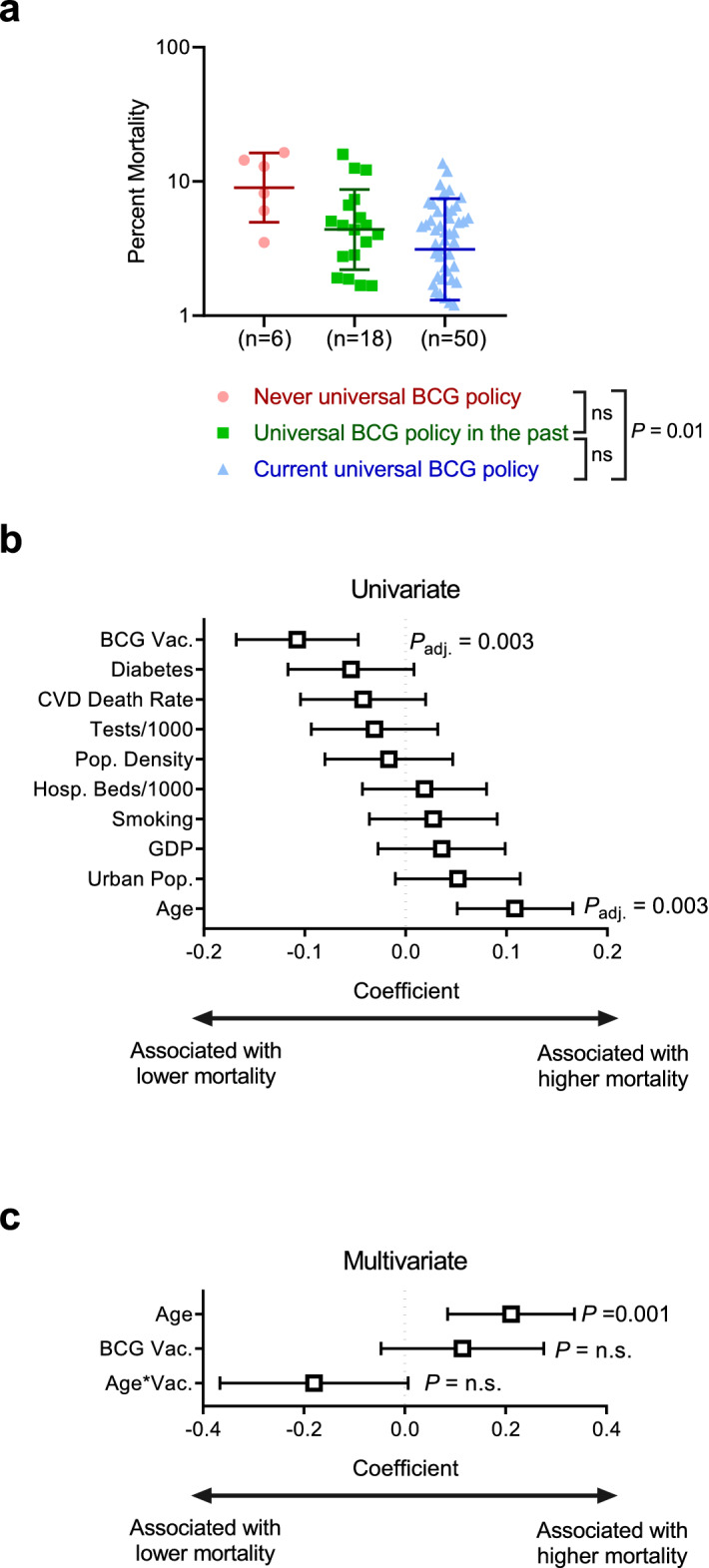
Distribution of COVID-19 mortality and BCG vaccination policy. (**a**) CoV-2 mortality is shown for all three groups of BCG policies (never (red), past (green) and current (blue) BCG vaccination policy). Data shown as median ± interquartile range. (**b**) Univariate analysis of CoV-2 mortality shows the coefficients and adjusted *p values* for BCG vaccination policy (BCG Vac.), diabetes, cardiovascular disease (CVD) death rate, tests per 1000 inhabitants, population density (pop. density), hospital beds per 1000 inhabitants, smoking rate, gross domestic product (GDP), urban population (urban pop.) and age. (**c**) Multivariate regression analysis of fraction of population over 65 and BCG vaccination policy with COVID19 percent mortality.

While we observed decreased COVID-19 spread rate in countries with universal BCG vaccination policies, this analysis is fundamentally limited by the extent to which countries are capable of testing for CoV-2 to quantify spread of COVID-19. Comparison of COVID-19 spread rate and rates of testing yielded a strong positive correlation of 0.68 (*p value* = 4.2 × 10^–11^) (Fig. 2a). CoV-2 testing rates were significantly different between countries with distinct BCG vaccination policies (Fig. 2b). When only including “high CoV-2 testing” countries with 10 or more tests per thousand, resulting in equivalent distribution of CoV-2 testing rates across countries with different BCG policies, COVID-19 spread rate is no longer associated with BCG policy (Fig. 2c). Consistent with this, multivariate analysis indicated testing rate, but not BCG vaccination policy, was associated with COVID-19 spread rate (Fig. 2d). To assess if additional factors could be confounding the analysis, we analyzed the association of population density (population/km^2^), fraction of population over 65, fraction of the population that lives in urban areas (urban pop.), per capita GDP, smoking rates, diabetes prevalence, and cardiovascular disease (CVD) related mortality with both BCG vaccination policy (Fig. 2e) and COVID-19 spread rate (Fig. 2f). Performing multivariate analysis of variables significantly associated with both BCG vaccination policy and COVID-19 spread rate indicated that testing rate and cardiovascular death rate were independently associated with increased COVID-19 spread rates (Fig. 2g). Percent COVID-19 mortality was significantly lower between countries that have a current universal BCG policy compared to countries that never had a universal BCG policy (*p value* = 0.01) (Fig. 3a). However, univariate analysis showed that age confounds the analysis (Fig. 3b). When adjusted for age the percent CoV-2 mortality is not different in countries with different BCG policies, though age remained statistically associated with percent mortality (*P* = 0.001) (Fig. 3c). Repeating these analyses using BCG vaccination policy as a binary variable, that is current universal policy *vs* no current universal policy, produced equivalent results with the exception that there was no longer a significant association between BCG vaccination policy and COVID-19 mortality (Supplementary Figure S2).

To further confirm our observations, we next repeated our analyses only in European countries, aiming to increase the homogeneity in population genetics and demographic characteristics to improve the quality of our comparisons. Upon initial analysis, we still observed distinct demographic differences between countries with and without current BCG vaccination policies (Supplementary Figure S3a). To address this, we performed K-means clustering based on demographic descriptors (excluding BCG vaccination policy), dividing Europe into two clusters (Supplementary Figure S3b). All countries in Cluster 2 had a BCG vaccination policy, but Cluster 1 had 3 countries without BCG vaccination policies (Belgium, Italy, and Netherlands). Analysis of only Cluster 1 found no demographic features (population density, fraction of population over 65, fraction of the population that lives in urban areas, hospital beds per capita, per capita GDP, smoking rates, diabetes prevalence, or cardiovascular disease related mortality) to be associated with BCG vaccination policy (Supplementary Figure S3c). In countries from Cluster 1, BCG vaccination policy was not associated with changes in COVID-19 spread rate (unadjusted *P* = 0.86) (Supplementary Figure S3d). Although no demographic features achieved *P* < 0.05 following correction for multiple comparisons, tests/1000 inhabitants and smoking had unadjusted *P* values < 0.05. Performing multivariate regression analysis using these two parameters indicated that both are likely independently associated with an increase in observed COVID-19 spread rate (Supplementary Figure S3e). Analyzing the univariate association of percent mortality from COVID-19 within countries from Cluster 1 revealed both GDP and Tests/1000 were significantly (*P*_adj._ < 0.05) associated with lower percent mortality, whereas age was only significant prior to correction for multiple comparisons (Supplementary Figure S3f). Multivariate regression analysis using three parameters indicated that smoking prevalence and tests/1000 inhabitants are likely independently associated with increased and decreased COVID-19 mortality, respectively (Supplementary Figure S3g).

## Discussion

Our analysis of COVID-19 spread rate in 74 countries that were stratified based on the prevalence of BCG vaccination (no policy, prior universal vaccination policy, or current universal vaccination policy) shows that, a priori, there is a correlation between current universal BCG vaccination policy and a lower COVID-19 spread rate and mortality. Interestingly, we note that testing rates (tests per thousand inhabitants) was positively correlated with incidence of CoV-2 infection. Taking in consideration this important bias, we stratified the data to compare the CoV-2 spread rate amongst countries with distinct universal BCG policy based on high CoV-2 testing rates, as defined by 10 or more tests per thousand inhabitants. The results show a lack of significant difference in COVID-19 spread rate in countries with current universal BCG vaccination policy and countries with never or in the past universal BCG vaccination policy.

Other variables tested in our analyses included GDP, median age, percent urban population, hospital beds per 1,000 inhabitants, smoking, population density, diabetes and CVD related deaths. GDP, median age, percent urban population and CVD related deaths were identified as additional confounders. We did not observe higher COVID-19 mortality when stratifying countries based on vaccine strain, however, that could at least partially be due to limited sample size (n = 44 countries).

Our study is limited by several critical considerations, including the variability and inaccuracies in reporting positive cases and reporting specific mortality associated with CoV-2 infection and lack of reporting on asymptomatic or minimally symptomatic cases. There could be also differences at the subnational level, for example as seen in India where most reported CoV-2 cases are from 5 states, which limits conclusions for the entire country. It is possible that there are also some countries that have subnational differences, for example in accessing healthcare, which could influence CoV-2 infection rates and are not accounted for in our analysis.

Another limitation could be the adherence and access to vaccination as per policy. BCG coverage data suggested a high coverage (> 90%) in most countries, therefore, countries are likely comparable in respect to BCG coverage. In our study, we address the impact of social and economic barriers in accessing care by including GDP and hospital beds per capita. Other studies used similar metrics, including the human development index (HDI), describing access to health and education^18^ and income^17^. Both studies show a correlation of universal BCG vaccination policies and the number of COVID-19 cases when controlling for HDI or income, however, neither of these studies took CoV-2 testing rates into consideration.

In our study, we try to take into account variables describing co-morbidities such as smoking rates, diabetes prevalence and cardiovascular disease related mortality. There are many more co-morbidities listed by the Center of Disease Control and Prevention, including cancer and obesity which could be additional confounding variables but haven’t been analyzed in our study.

Differences in the virus strains could also confound our analyses, which is supported by a recent study showing that individuals infected with the spike protein variant G614 have higher viral loads^19^. Suggested polymorphisms in ACE2 itself could further confound our analysis^20^. Interestingly, BCG vaccination status did not correlate with COVID-19 mortality even within the same geographical location as shown in two recent studies^21,22^. It is also possible that countries with higher temperatures have lower transmission rates as suggested in a recently published study, however, this is also an epidemiological study that is confounded by multiple variables^23^. Finally, time since first exposure rates, as well as population adherence to social distancing measures, may also influence the evolving COVID-19 data collection. To date, social distancing remains critical in slowing down the number of new cases over time^24^.

In our study, we provide evidence that CoV-2 testing rates is a major confounder in the association between BCG vaccination policy and COVID-19 spread rate. Our study cautions against epidemiological studies showing correlation between BCG vaccination policy and COVID-19 spread rate. Currently conducted clinical trials are necessary to provide scientific data to evaluate the hypothesis that the BCG vaccine is effective to lower COVID-19 incidence and severity.

## Supplementary information

Supplementary Information
