# Supplementary material for: Protection against SARS-CoV-2 by BCG vaccination is not supported by epidemiological analyses
Source: Sci Rep. 2020 Oct 27;10:18377. doi: 10.1038/s41598-020-75491-x (PMC7591473; doi:10.1038/s41598-020-75491-x)
Supplement: Supplementary file 1 — Supplementary Information [file 41598_2020_75491_MOESM1_ESM.pdf]

Supplementary data file

Protection against SARS-CoV-2 by BCG vaccination is not supported by epidemiological analyses

Janine Hensel[1#], Kathleen M. McAndrews[1#], Daniel J. McGrail[2#], Dara P. Dowlatshahi[1], Valerie S. LeBleu[1,3] and Raghu Kalluri[1,4,5,*]

[1]Department of Cancer Biology, Metastasis Research Center, University of Texas MD Anderson Cancer Center, Houston, TX, USA.

[2]Department of Systems Biology, University of Texas MD Anderson Cancer Center, Houston, TX, USA.

[3]Feinberg School of Medicine, Northwestern University, Chicago, IL, USA.

[4]Department of Bioengineering, Rice University, Houston, TX, USA.

[5]Department of Molecular and Cellular Biology, Baylor College of Medicine, Houston, TX, USA.

[#]Co-first authors
*Corresponding author. Email: rkalluri@mdanderson.org

Supplementary Figure 1

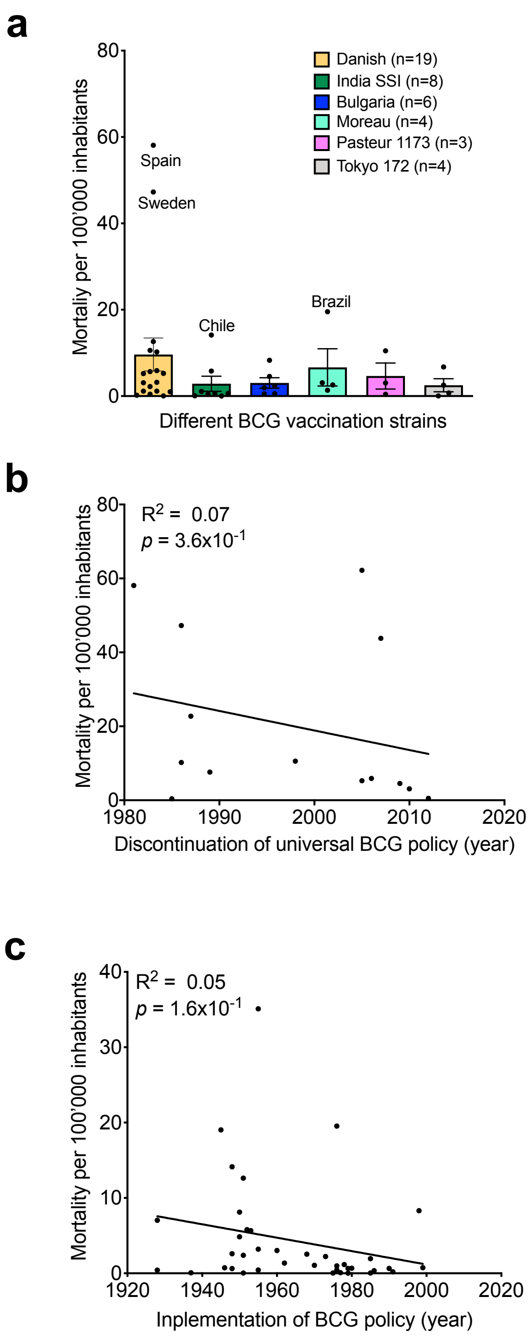

Supplementary Figure 1. COVID-19 mortality is not correlated with discontinuation or implementation of universal BCG policy.
(a) CoV-2 mortality per 100,000 inhabitants shown as mean ± SEM for different BCG strains. **(b)** Correlation graph showing mortality per 100,000 inhabitants and the time since discontinuation of universal BCG policy.
(c) Correlation graph showing mortality per 100,000 inhabitants and the time since implementation of universal BCG policy.

Supplementary Figure 2

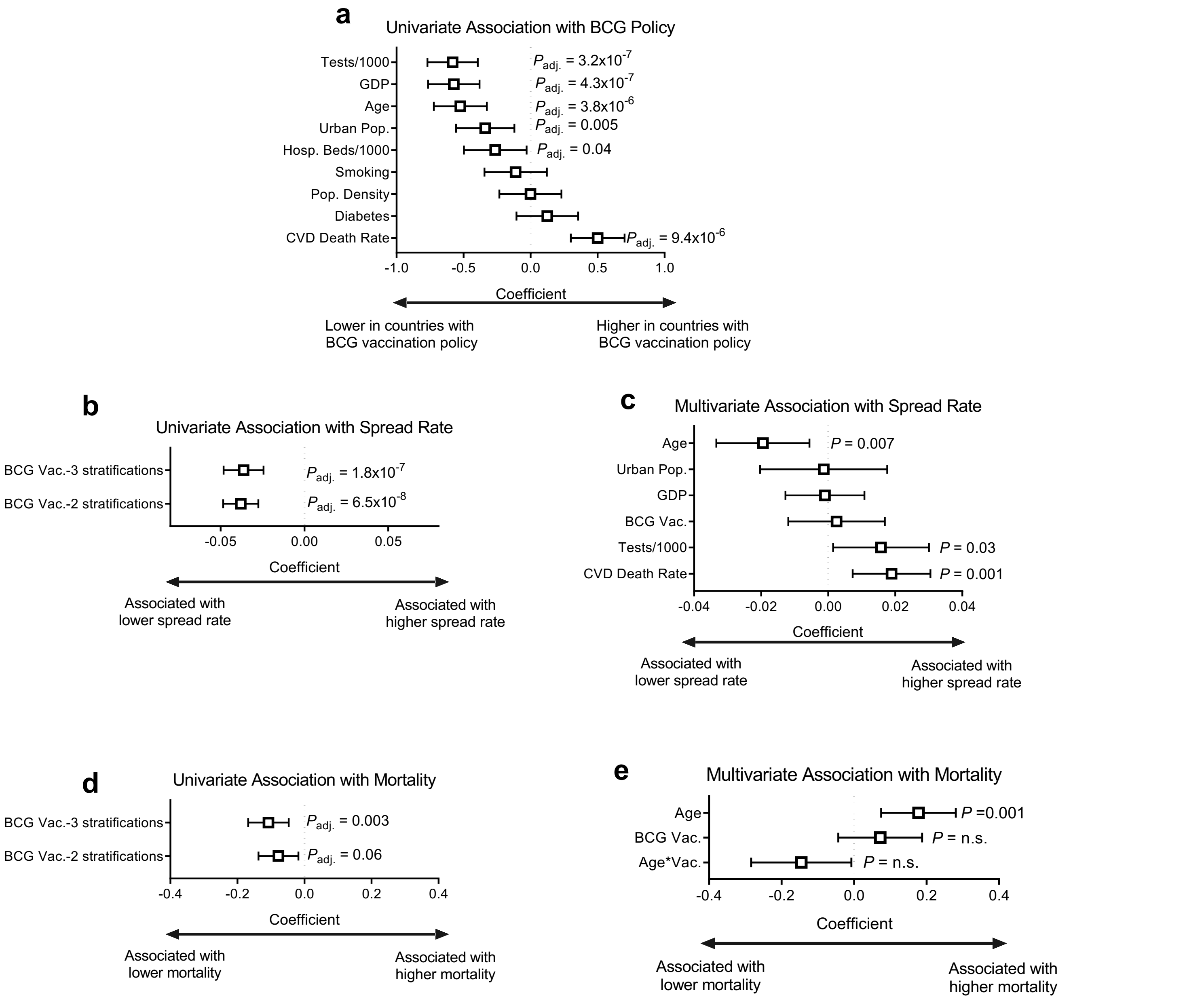

Figure S2. Analysis of associations with BCG vaccination policies using vaccination policy as a binary variable (no universal policy *vs* universal policy).

(a) Univariate analysis of associations with BCG vaccination policy comparing countries with current universal vaccination policy *vs* no current policy shows the coefficients and adjusted *p-values* for cardiovascular disease (CVD) death rate, diabetes population density (pop. density), smoking rate, urban population (urban pop.), hospital beds per 1000 inhabitants, gross domestic product (GDP), age and tests per 1000 inhabitants.

(b) Comparison of univariate analysis for the associations with CoV-2 spread rate when treating BCG vaccination policy as 3 distinct strata (never universal policy, prior universal policy, current universal policy) with 2 distinct strata (no universal policy *vs* universal policy).

(c) Multivariate regression analysis of CoV-2 spread rate using BCG vaccination policy as a binary variable (no universal policy *vs* universal policy).

(d) Comparison of univariate analysis for the associations with CoV-2 mortality when treating BCG vaccination policy as 3 distinct strata (never universal policy, prior universal policy, current universal policy) with 2 distinct strata (no universal policy *vs* universal policy).

(e) Multivariate regression analysis of fraction of population over 65 and BCG vaccination policy with COVID19 percent mortality using BCG vaccination policy as a binary variable (no universal policy *vs* universal policy).

Supplementary Figure 3

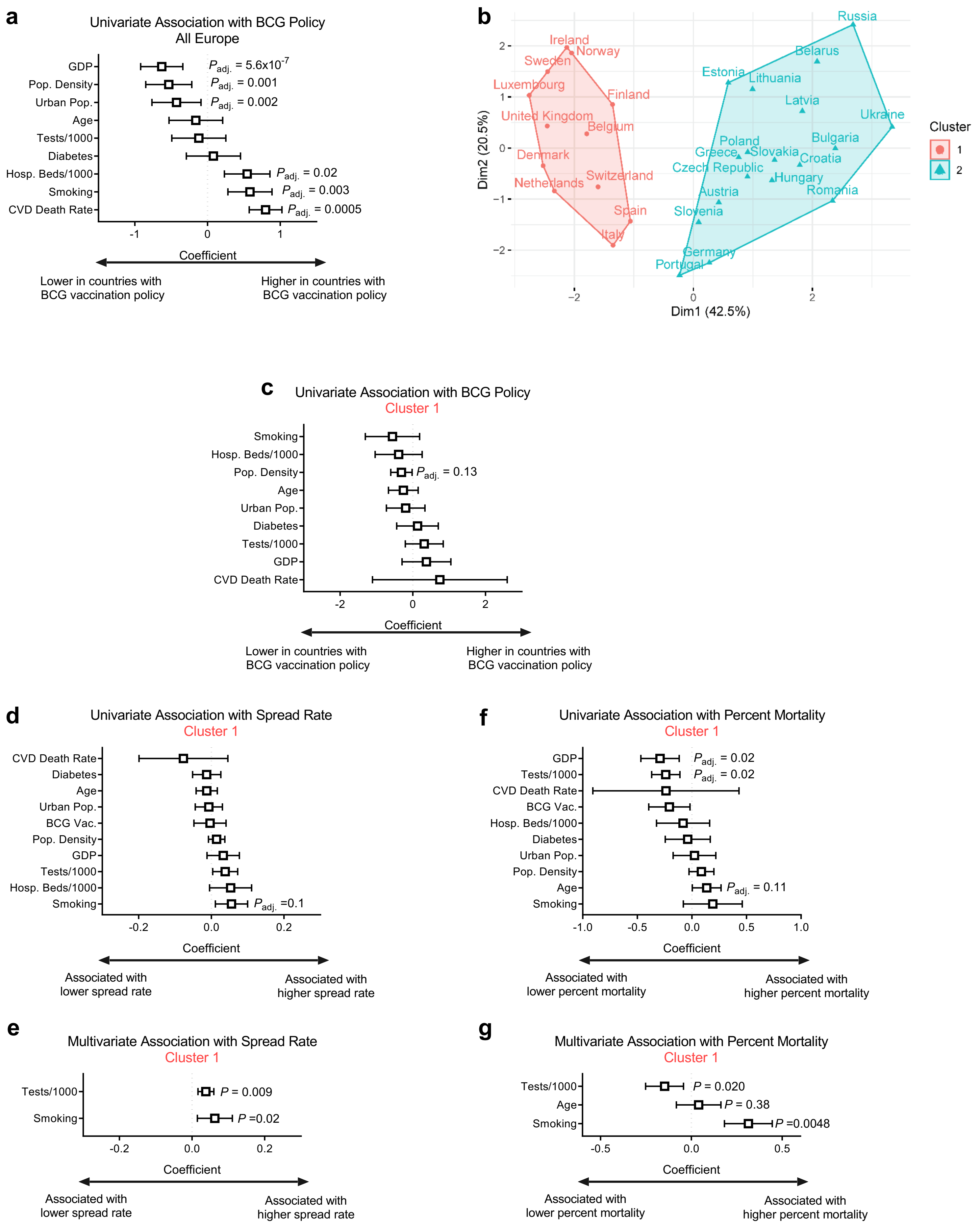

Supplementary Figure 3. Analysis of BCG vaccination policy in European countries.
(a) Univariate analysis of associations with BCG vaccination policy showing the coefficients and adjusted *p-values* for cardiovascular disease (CVD) death rate, diabetes population, density (pop. density), smoking rate, urban population (urban pop.), hospital beds per 1000 inhabitants, gross domestic product (GDP), age and tests per 1000 inhabitants.
(b) PCA plot and K-means clustering of European countries based on demographic descriptors shown in S2a.
(c) Univariate analysis of associations with BCG vaccination policy in cluster 1 (red) showing the coefficients and adjusted *p-values* for cardiovascular disease (CVD) death rate, diabetes population, density (pop. density), smoking rate, urban population (urban pop.), hospital beds per 1000 inhabitants, gross domestic product (GDP), age and tests per 1000 inhabitants.
(d) Univariate analysis of associations with COVID-19 spread rate in cluster 1 (red) showing the coefficients and adjusted *P*-values for cardiovascular disease (CVD) death rate, diabetes population, density (pop. density), smoking rate, urban population (urban pop.), hospital beds per 1000 inhabitants, gross domestic product (GDP), age and tests per 1000 inhabitants.
(e) Multivariate regression analysis of COVID-19 spread rate in cluster 1 (red) using variables with unadjusted *P*-values<0.05 from univariate analysis showing the coefficients and *P*-values, specifically tests per 1000 inhabitants and smoking prevalence.
(f) Univariate analysis of associations with COVID-19 percent mortality in cluster 1 (red) showing the coefficients and adjusted *P*-values for cardiovascular disease (CVD) death rate, diabetes population, density (pop. density), smoking rate, urban population (urban pop.), hospital beds per 1000 inhabitants, gross domestic product (GDP), age and tests per 1000 inhabitants.
(g) Multivariate regression analysis of COVID-19 percent mortality in cluster 1 (red) using variables with unadjusted *P*-values<0.05 from univariate analysis showing the coefficients and *P*-values, specifically tests per 1000 inhabitants, smoking, and hospital beds per 1000 inhabitants.

Table S1. BCG vaccination policy and tuberculosis incidence rates by countries.

Country	TB incidence/100000	TB incidence	Current vaccination
Belgium	9	1100	Never
Canada	5	1800	Never
Italy	6	3500	Never
Lebanon	13	750	Never
Netherlands	6	980	Never
USA	3	10000	Never
Andorra	7	5	Past
Australia	6	1400	Past
Austria	8	650	Past
Czechia	5	550	Past
Denmark	6	340	Past
Ecuador	52	8400	Past
Finland	6	310	Past
France	8	5300	Past
Germany	8	6500	Past
Israel	4	320	Past
Luxembourg	6	34	Past
Norway	6	330	Past
Slovakia	7	350	Past
Slovenia	7	150	Past
Spain	12	5500	Past
Sweden	9	900	Past
Switzerland	7	610	Past
UK	10	6600	Past
Belize	25	90	Present
Bhutan	155	1200	Present
Botswana	356	8000	Present
Burundi	122	14000	Present
Cambodia	380	59000	Present
Dominica	11	8	Present
Eritrea	65	3400	Present
Fiji	51	450	Present
Gambia	174	3500	Present
Greenland	164	92	Present
Hong Kong	71	5200	Present
Laos	182	12000	Present
Mongolia	428	13000	Present
Mozambique	551	154000	Present
Myanmar	365	197000	Present
Namibia	489	12000	Present
Papua New Guinea	432	33000	Present

Rwanda	56	6600	Present
Saint Kitts and Nevis	5	3	Present
Saint Lucia	9	16	Present
Seychelles	10	9	Present
St. Vincent Grenadines	7	8	Present
Syria	20	3200	Present
Timor-Leste	498	5900	Present
Uganda	202	79000	Present
Vietnam	137	128000	Present
Afghanistan	189	61000	Present
Albania	19	550	Present
Algeria	75	30000	Present
Angola	370	93000	Present
Argentina	25	11000	Present
Armenia	41	1200	Present
Azerbaijan	69	6800	Present
Bangladesh	225	362000	Present
Barbados	unknown	unknown	Present
Belarus	55	5200	Present
Benin	60	6600	Present
Bolivia	117	13000	Present
Bosnia and Herzegovina	37	1400	Present
Brazil	41	84000	Present
Bulgaria	24	1700	Present
Burkina Faso	52	9400	Present
Cameroon	212	49000	Present
Chad	152	21000	Present
Chile	16	3000	Present
China	67	918000	Present
Colombia	31	15000	Present
Congo	379	18000	Present
Congo Dem Rep	324	250000	Present
Costa Rica	11	530	Present
Croatia	13	560	Present
Cuba	7	800	Present
Djibouti	378	3400	Present
Dominican Republic	60	6300	Present
Egypt	15	13000	Present
El Salvador	43	2700	Present
Equatorial Guinea	172	1500	Present
Estonia	18	240	Present
Ethiopia	192	191000	Present
Gabon	465	8000	Present
Georgia	99	4000	Present

Ghana	160	44000	Present
Greece	5	490	Present
Guatemala	25	4200	Present
Guinea	177	22000	Present
Guinea-Bissau	373	6900	Present
Guyana	93	710	Present
Haiti	194	21000	Present
Honduras	43	3500	Present
Hungary	9	920	Present
India	217	2840000	Present
Indonesia	395	1020000	Present
Iran	16	13000	Present
Iraq	43	16000	Present
Ireland	7	340	Present
Jamaica	5	130	Present
Japan	17	21000	Present
Jordan	7	530	Present
Kazakhstan	89	16000	Present
Kenya	233	107000	Present
Kuwait	22	860	Present
Kyrgyzstan	144	8500	Present
Latvia	41	800	Present
Liberia	308	14000	Present
Libya	40	2500	Present
Lithuania	56	1600	Present
Madagascar	236	57000	Present
Malaysia	89	27000	Present
Maldives	53	190	Present
Mali	57	10000	Present
Malta	9	37	Present
Mauritania	107	4300	Present
Mauritius	22	280	Present
Mexico	21	27000	Present
Moldova	152	6200	Present
Morocco	107	37000	Present
Nepal	156	44000	Present
New Zealand	7	340	Present
Nicaragua	51	3100	Present
Niger	95	19000	Present
Nigeria	322	586000	Present
North Macedonia	13	270	Present
Oman	8	380	Present
Pakistan	270	510000	Present
Panama	50	2000	Present

Paraguay	41	2700	Present
Peru	119	37000	Present
Philippines	322	324000	Present
Poland	19	7200	Present
Portugal	23	2400	Present
Qatar	34	760	Present
Romania	84	160000	Present
Russia	80	115000	Present
Saudi Arabia	12	3800	Present
Senegal	139	21000	Present
Sierra Leone	307	20000	Present
Singapore	44	2500	Present
Somalia	274	30000	Present
South Africa	834	454000	Present
Sri Lanka	65	13000	Present
Sudan	88	35000	Present
Taiwan	unknown	unknown	Present
Tanzania	306	164000	Present
Thailand	172	117000	Present
Togo	52	3800	Present
Tunisia	37	4200	Present
Turkey	18	14000	Present
UAE	2	140	Present
Ukraine	91	41000	Present
Uruguay	30	1000	Present
Uzbekistan	79	24000	Present
Venezuela	29	8900	Present
Zambia	391	63000	Present
Zimbabwe	242	38000	Present

Table S2. BCG coverage in percent (%)

Country	Current vaccination	BCG coverage in %
Ukraine	Present	15-45
Ethiopia	Present	69
Haiti	Present	70
UK	Past	75
Sudan	Present	77
Colombia	Present	80-90
Czechia	Past	80
Kenya	Present	80
Peru	Present	80
Uganda	Present	85
Nigeria	Present	85
Senegal	Present	85
El Salvador	Present	85.4
Angola	Present	90

Congo	Present	90
Estonia	Present	90
Sierra Leone	Present	90
Israel	Past	>90
Romania	Present	>90
South Africa	Present	90.5
Indonesia	Present	92.2
Vietnam	Present	93.7
Poland	Present	93.7
Ireland	Present	94
Turkey	Present	94
Bhutan	Present	95
Rwanda	Present	95
Argentina	Present	95
Mexico	Present	95
Tunisia	Present	>95
North Macedonia	Present	95.36
Slovenia	Past	96
Latvia	Present	96.1
Brazil	Present	96.9
Greenland	Present	97
Bosnia and Herzegovina	Present	97
Singapore	Present	97
Chile	Present	97.8
Finland	Past	98
Belarus	Present	98
Bulgaria	Present	98
Saudi Arabia	Present	98
Japan	Present	98.1
Mongolia	Present	98.6
Croatia	Present	98.6
Gambia	Present	99
India	Present	99
Thailand	Present	99
Iran	Present	99.5
Uruguay	Present	99.9
Tanzania	Present	100
